# Supplementary material for: Are carbon emissions trading and green financial instruments synergistic? -Comprehensive quantitative research based on content analysis
Source: PLoS One. 2024 Mar 7;19(3):e0298601. doi: 10.1371/journal.pone.0298601 (PMC10919601; doi:10.1371/journal.pone.0298601)
Supplement: S1 Data — (ZIP) [file pone.0298601.s001.zip › Data/地级市Z_F工作报告.zip!/╣π╢1⁄2/║╙╘┤╩╨/└┤╫╘╣1⁄2╓┌║┼í╛┬φ┐╦╩2╛▌═°í┐_2009.docx]

各位代表：

我代表市人民政府向大会作政府工作报告，请予审议，并请各位政协委员和其他列席人员提出意见。

一、过去一年工作回顾

２００８年，是我市经济社会发展遇到突出困难和重大挑战的一年，也是我市经受严峻考验取得新的可喜成绩的一年。一年来，我市先是在国家实施从紧货币政策的大背景下，碰到了人民币持续升值、原材料价格上涨、企业融资困难、生产成本上升等突出困难；接着又在国际金融危机、全球经济衰退的大背景下，遇到了出口订单减少、企业亏损增加、固定资产投资和利用外资下降、房地产市场低迷等突出困难；同时，在我市被省列为生态发展区的大背景下，承受了节能减排力度加大，一批在县区工业增长中扮演重要角色的小钢铁、小水泥等高能耗、高排放企业被关停的巨大压力。面对复杂的国际国内经济形势和各种困难挑战，在省委、省政府和市委的正确领导下，在市人大及其常委会和市政协的监督支持下，我们坚持以邓小平理论和“三个代表”重要思想为指导，以科学发展观统领经济社会发展全局，认真贯彻落实党中央、国务院和省委、省政府一系列决策部署以及市委五届四次、五次全会精神，坚定信心，沉着应对，迎难而上，咬定发展第一要务不放松，注重抢抓各种机遇，不断创新工作措施，着力破解发展难题，依期完成了经济社会发展主要预期目标和任务。

（一）国民经济保持平稳较快增长。２００８年，全市生产总值３９４．１３亿元，比上年增长（下同）１０．６％。其中第一产业增加值５２．７６亿元，第二产业增加值２１６．５７亿元，第三产业增加值１２４．８０亿元，分别增长５．４％、１２．８％和９．１％。三大产业结构由上年的１３．４∶５３．４∶３３．２调整为 １３．４：５４．９：３１．７。 人均生产总值达１３８６０元，增长 ８．９％。 全社会工业增加值１９９．７５亿元，规模以上工业增加值１８３．４６亿元，分别增长１５．６％和１６．５％；地方财政一般预算收入１７．６１亿元，增长１６．２％；完成税收总收入３６．４５亿元，增长２２．３％；社会消费品零售总额１２３．９６亿元，增长１９．１％；全社会固定资产投资１７７．１８亿元；外贸出口额１２．４７亿美元，增长３９．３％，增幅居全省第一；实际利用外资３．２２亿美元熏增长１０．１％。城镇年末登记失业率控制在３．０８％；农村居民人均纯收入４７２９元，城镇居民人均可支配收入１１３４３元，分别增长６．７％和７．０％；人口自然增长率７．５‰；居民消费价格指数１０６．４％。节能减排任务全面完成。 （二）承接产业转移取得重大进展。中山（河源）产业转移园经过竞标成为全省首批示范性产业转移园，并成功获得省政府５亿元竞争性扶持资金，规划面积扩大到５０平方公里，目前正在进行三期征地工作。全市产业转移园全部列入省认定范围。先后在广州、深圳和东莞召开投资环境推介会，举办了２００８年中国ＣＤＭＡ产业发展论坛，组团参加了省首届新粤商大会和第五届“中博会”、２００８粤港—欧洲（西班牙）经济技术贸易合作交流会和第五届“山洽会”。全年新签约进园区项目６１个，投资总额６７．８２亿元；园区新开工项目１１２个，投资额９８．３９亿元；竣工项目８０个，投资额８２．４３亿元。我市手机基地被认定为广东省手机产业集群升级示范区，国家通讯终端产品质量监督检验中心、移动通讯产学研战略联盟等技术服务平台加快建设，市高新区手机年产量达７０６万台，产值４０．５亿元。和平钟表产业、紫金光学眼镜产业、东源新型材料产业、连平绿色食品加工产业、源城和龙川电子电器制造产业等主导产业培育也取得新进展。制定扶持中小企业加快发展行动方案和配套政策，出台“暖企工程”７条措施，积极帮助企业克服困难，营造与企业同舟共济、共渡难关的良好氛围。产业转移园对经济发展的贡献进一步显现。全市“一区六园”完成规模以上工业总产值、工业增加值和入库税收分别为３７４．４１亿元、１０５．１５亿元和 ６．３１亿元， 分别增长１９．２％、２０．４％和４５．１％，对全市经济增长和税收增长的贡献率分别达４２．９％和２９．５％。

（三）城乡规划和重大项目建设拉开新格局。城乡规划全面铺开。全面启动了《河源市城市总体规划修编》；新一轮土地利用总体规划大纲修编扎实推进；灯塔盆地开发的规划论证工作取得阶段性成果；完成了《河源市中心城区概念规划》、《河源市城区干线道路网规划》、《河源市东城中片区、东片区及西片区控制性详细规划》、《河源市近期建设规划》等四个重大规划；基本完成了《河源市公路网规划》和 《河源市水路岸线及港口总体布局规划》的编制工作；市区有关区域和相关专项控制性详细规划等编制工作取得阶段性成果。争取省批准新增紫金临江镇为中心镇。１８个省级中心镇近期建设规划、２０个重点镇总体规划和１５０个村庄规划试点的规划编制工作扎实推进。重大项目建设取得可喜突破。万绿湖直饮水工程前期工作取得重大进展，市政府分别与深圳、东莞市政府签订了合作框架协议，省发改委正主导开展项目可行性咨询论证；河源电厂一期工程顺利推进，１号机组已正式投产发电；提前启动《河源市“十二五”电网专项规划》，加快推进“１·８·２８”电网工程和农网改造工程。广河高速公路已于去年全面开工，汕湛高速公路河紫段、汕昆高速公路龙川至连平段已完成工程可行性论证评审，大广高速公路连平段开工在即，粤湘高速公路连平段正进入设计阶段，龙川至汕尾铁路纳入了国家和省的规划；市区七条市政重点道路建设的前期工作全面启动；改造国道１３公里、省道１１０．５公里、县乡公路９９公里。

（四）现代农业和新农村建设稳步推进。采取一系列措施促进农业增效、农民增收和农村发展。全市农业总产值８６．１４亿元，增长５．２％。支农惠农政策全面落实，发放种粮综合直补１．２６亿元、能繁母猪补贴６８６万元。全面完成了省下达的粮食考核指标。市、县财政安排５０００万元贷款贴息重点支持发展农业特色产业。争取国家连续三年每年支持４０００万元规划建设１５万亩、改造２万亩的东江上游特色水果产业带。成功举办河源市首届现代生态农业推介暨农产品展销会。全市新增省、市级农业龙头企业１５家，辐射带动１５．３万农户，促进农户户均增收２３００元。创办新农村建设示范点９８个。加快农村发展体制、机制创新，完成了畜牧兽医、种子和水利工程管理体制改革。启动“农村清洁工程”试点工作。建成城市防洪堤１１３．４３公里，完成中型水库加固工程２宗、小型水库除险加固工程４８宗、省人大农村机电排灌议案工程３０宗，防灾减灾工作连续五年被省政府评为一等奖。在全省率先启动小流域综合治理试点工作。新一代天气雷达项目建设进展顺利，气象监测、预报、预警服务体系进一步完善。筹资６０００万元在市高新区筹建富民工业园Ｃ区；全面落实水库移民后期扶持政策，加快库区移民公共基础设施建设，移民人均年纯收入增长５．３％。“千村脱困”政策全面落实，１１１７个贫困村共获得投资分红款２６００多万元。农村年人均纯收入１５００元以下的贫困户和贫困人口减少到６．６７万户、２１．８５万人，分别下降６．７％和６．９％。

（五）以旅游业为重点的第三产业发展加快。市政府与省旅游局签订了《共建“广东省生态旅游示范区”合作协议》，全力推动旅游业发展和转型升级。通过召开全市旅游景区景点建设现场会，成立市规划委员会旅游规划分会，制定出台系列管理规定等，进一步加强了旅游景区景点规划建设和管理。按五星级标准建设的万绿湖国际会议度假中心施工进展顺利，市区商业中心、恐龙文化公园、一批五星级酒店及大型旅游项目招商步伐加快。加强旅游宣传促销，举办了第二届广东自驾旅游节暨第五届河源市客家文化旅游节系列活动，加强了粤赣闽三省十个客家地级市区域旅游合作。全年接待入市游客７２３．１万人次，旅游总收入３１．０２亿元，分别增长１６．２％和１５．１％。商贸流通、运输仓储、连锁经营、物流配送、食宿餐饮以及信息中介、社区服务等服务业进一步发展。

（六）生态建设和环境保护力度加大。扎实推进林业生态市建设。全市共筹资近６亿元，全面启动林业体制改革。进一步规范速生丰产桉种植和木材加工企业管理。完成造林作业面积１９．０４万亩，新营造生物防火林带３０６．２公里、面积３９３．７公顷。环保基础设施建设取得新突破。市生活污水处理厂过江管网正在加快铺设，市城南污水处理厂成为全省产业转移园首个建成运营的污水处理厂，县区１１座污水处理厂全面动工，七寨生活垃圾卫生填埋场建设稳步推进。完成国控重点污染源在线自动监控设备安装，并与省环保局正式联网。对高耗能、高排放行业进行专项整治，关停、淘汰落后炼钢产能２２５．５万吨、炼铁产能３万吨，关停小水泥企业２家。开展整顿规范矿产资源开发秩序 “回头看”活动，坚决查处违法采矿死灰复燃现象，矿产资源开发进一步规范。对万绿湖水面养殖和污染环境的规模养猪场进行专项清理整顿，组织整治市区东埔河专项行动，并取得明显阶段性成效。

（七）财税金融运作稳健富有成效。在税收政策调整、税源发生变化、税收增长难度明显加大的情况下，积极采取应对措施，强化税源管理，加强有效引导，挖掘增收潜力，财税增收保持了较高的增幅，国地两税收入分别增长３３．３％和１２．７％。面对各种建设任务繁重、改善民生要求不断提高、新增硬性支出项目越来越多，而资金严重短缺的困难，注重开源节流、科学理财，基本实现了保运转、保发展、保民生的目标。用足用好上级政策，积极争取上级财力支持，去年争取省级资金支持比上年增加１２亿元 （含省示范性产业转移园竞争性扶持资金）。加强土地储备和开发工作，提高经营城市水平。加强审计监督，对县区委书记、县区长同时进行经济责任审计的经验在全国推广。加强与金融机构协作，完善政银企沟通合作机制，积极争取金融部门支持，市政府与国家开发银行广东省分行签订了资金额度４８亿元的合作协议，促成广东发展银行河源分行与我市中小民营企业签订了１１亿元的银企合作协议。获得工商银行河源分行、农业银行河源分行、中国银行河源分行、农业发展银行河源分行等多家金融机构的项目贷款。注重统筹兼顾，科学调度资金，有力地支持了全市工业园区、基础设施、环境保护、重点项目和民生项目的建设。去年用于推进“十项惠民工程”和“十件实事”资金达１８．１亿元，民生方面的资金投入比上年增长２１．９％；安排涉农资金６．６２亿元；安排社会保障和就业资金９．９亿元，增长１７．７％；消化上年度贷款３．５亿元，偿还历史债务２．２亿元；完成了路桥收费站的撤并整合，核准了交通建设还贷基数，获得６．５亿元还贷资金，有效解决了长达十几年的交通建设历史欠账；市级一般预算补助县区支出１．８２亿元，比上年增加３１．５％。全市金融业健康发展，金融机构存款余额３３４．８亿元，贷款余额２０１．３亿元，分别增长１６．８％和１９．６％。金融改革继续推进，城市信用社退市工作进展顺利，农村信用社体制改革取得新突破。

（八）社会事业和民生建设成效显著。扎实推进“十项惠民工程”。全市教育事业有了长足发展，新增高中阶段学位９７４６个，高中阶段毛入学率达６９．７６％，提高了７个百分点；城乡免费义务教育政策全面落实；全面提高教师待遇，在全省率先实现了县域内中小学教师平均工资水平与当地公务员基本持平。统筹城乡就业，新增就业岗位３．９１万个，新增转移农村劳动力６．６７万人。全市新增参加养老、失业、医疗、工伤、生育各险种５１万人次，其中启动了城镇居民基本医疗保险，参保人数达２５万多人，全面完成了省下达的参保任务。城乡低保居民实现应保尽保，低保救济面达７％。稳步推进新型农村合作医疗制度，参合率达９３％。全力开展抢险救灾工作，４７０９户全倒户元旦前全部迁入新居。募集资金３６３６万元，积极支援汶川灾区抗震救灾工作。全面开展计划生育创“两无”活动，计划生育率９１．４６％，三类县、镇转化工作取得重大进展，全面完成了省政府下达的计划生育任务。加强了产品质量和食品安全监督，“五小”专项整治经验在全省推广。加强重大疾病和公共卫生安全防控，扎实做好手足口病防治和问题奶粉处置工作。采取有力措施打击“三违四抢”行为，拆除市区及高新区征地控制范围违法违章建筑９．５４万平方米。开展交通秩序及运输市场秩序联合整治 “百日行动”，并出台了严格的考核问责制度，道路交通和运输市场秩序明显好转。组织开展“清洁河源百日大行动”，理顺了市区环卫管理体制，市区和各县城的环境卫生明显改善。扎实有效推进应急管理工作，认真开展安全生产“隐患治理年”活动，各类安全生产事故起数、死亡人数、受伤人数分别下降１９．６％、２０．９％和９．８％，直接经济损失减少２５．１％。公安“三基”工程扎实推进，政法系统基层基础建设不断加强。深入开展“平安奥运”等专项行动，刑事立案数下降７．８％，破案率上升１０．７个百分点，其中“两抢一盗”立案数下降７．９％。积极推进“六好”平安和谐社区建设，双拥、信访、打私、人民调解、法制宣传教育和法律援助工作有效推进。举办了建市２０周年庆典系列活动和中国首届客家文化节，成功获得了第２３届世客会承办权。举行了第二届市运会，激流回旋运动项目获得１个世界冠军、７个全国冠军，实现运动员参加奥运会零的突破。投入资金９．４亿元，完成年内承诺办好的“十件实事”。其中，全面完成９５个行政村６００公里水泥硬底化建设，全面完成３５个社区居委会办公用房建设；市财政拨出专款４３０万元、社会筹集资金约５００万元，设立了市扶困助学基金；总投资４亿多元的市人民医院新院和市妇幼保健院新院顺利竣工，市中医院搬迁改建顺利启动；投入３９００万元对２８所农村医疗机构进行标准化建设；筹资９７２５万元，解决了农村２０．５万人饮水不安全问题；市民一度反映较多的市区用水问题得到较好解决；市区新增高中阶段学位３０７５个、义务教育阶段学位１８０２个，首期投资３．３亿元的市工业技术学校正式动工；市广播电视中心、市人防应急指挥中心将在今年第一季度竣工，市博物馆新馆建设和龟峰塔修缮工程顺利进行；客家文化公园、市图书馆新馆建设前期工作已全面启动；培训农村劳动力转移就业４．０８万人；完成１３２２４户贫困户 （其中移民７２２４户）危旧房改造任务；提高城乡社会保障水平任务全面完成；市直行政事业单位干部职工年人均增加津补贴５０００元，住房货币补贴全面实施，新建经济适用房和新增廉租房任务如期完成。全市１４０５个村（居）委会换届选举顺利完成。进一步加强了民族宗教、外事侨务、统计、物价、供销、人事编制和信息化工作，科普、知识产权、档案、史志、妇女、儿童、老龄、残疾人、民兵预备役等社会各项工作取得新的成绩。与此同时，市政府自身建设不断加强，科学决策和依法行政水平进一步提高，行政审批电子监察系统实现向县区延伸，行政复议和行政执法监察力度进一步加大，政府廉政建设、行政效率和执行力得到增强。认真执行市人大及其常委会各项决议并向其报告工作，坚持向人民政协通报情况，自觉接受监督。全年共办理市人大代表议案１件、建议９９件，市政协提案１１４件，办结率和办理质量都有新的提高。

各位代表，过去一年所取得的成绩，是省委、省政府和市委正确领导的结果，是市人大、市政协和社会各界监督支持的结果，是深圳、中山等兄弟市和省直挂钩帮扶单位、海内外投资者以及港澳台同胞、海外侨胞大力支持的结果，是全市人民艰苦奋斗的结果。在此，我代表市人民政府，向各位人大代表和政协委员，向长期关心支持我市发展的各级领导和各界朋友，向积极参与河源建设的海内外客商和外来务工人员，向驻河源解放军和武警、消防官兵，向各民主党派、人民团体、社会各界人士，向全市广大干部群众，表示衷心感谢并致以崇高敬意！

我市在国际经济危机、国内经济增长下滑的大环境下取得以上成绩，殊为不易。我们深切体会到：只有狠抓发展第一要务不动摇，坚定不移走新型工业化道路，以工业化带动农业产业化、城镇化和旅游产业化，千方百计做大经济蛋糕，才能从根本上改变河源经济总量小、财力薄弱的现状；只有注重打基础、谋长远不动摇，坚定不移践行“既要金山银山、更要绿水青山”的发展理念，统筹当前与长远的关系，才能不断提升河源发展水平，增强发展后劲，实现科学发展、协调发展；只有坚持解放思想、真抓实干不动摇，以只争朝夕的紧迫感、知难而进的勇气、脚踏实地的作风，善于抢抓机遇谋求发展，善于结合实际创造性开展工作，善于灵活应对各种困难和挑战，善于抓重点破难点出亮点，才能推动各项工作取得实实在在的进展；只有坚持依法行政、规范办事不动摇，主动接受市人大及其常委会、市政协和社会各界的监督，高度重视政府自身建设，自觉廉洁从政，对事关河源核心利益和发展大局的事情敢负责任敢抓敢管，才能不断提高科学决策水平和政府执行力；只有坚持执政为民、关心民生不动摇，扎实推进各项惠民工程，实实在在为百姓办实事谋福祉，不断提升老百姓幸福感，才能赢得人民群众的信赖、拥护和支持。

我们也清醒地看到，我市经济社会发展还存在不少困难和问题。主要是：经济基础仍然薄弱，经济总量仍然太小；产业竞争力不够强，经济效益不够好；招商选资力度不够大，引进大项目好项目不够多；城乡发展不平衡，农民持续增收难度增大；财源税源不足，财政收支矛盾仍然突出；人才不足，自主创新能力较弱；生态环保、社会保障、社会治安、安全生产、城市管理等方面还存在一些薄弱环节；机关作风不够扎实，政府执行力有待进一步提高。对这些困难和问题，我们将采取有效措施，认真加以解决。
